# Chalcogen Effects in the Photophysical Properties of Dimethylamino-1,8-naphthalimide Dyes Revealed by DFT Investigation

**DOI:** 10.1021/acs.jpca.2c03950

**Published:** 2022-07-27

**Authors:** Marta Erminia Alberto, Bruna Clara De Simone, Tiziana Marino, Marirosa Toscano, Nino Russo

**Affiliations:** Dipartimento di Chimica e Tecnologie Chimiche, Università della Calabria, 87036 Rende (CS), Italy

## Abstract

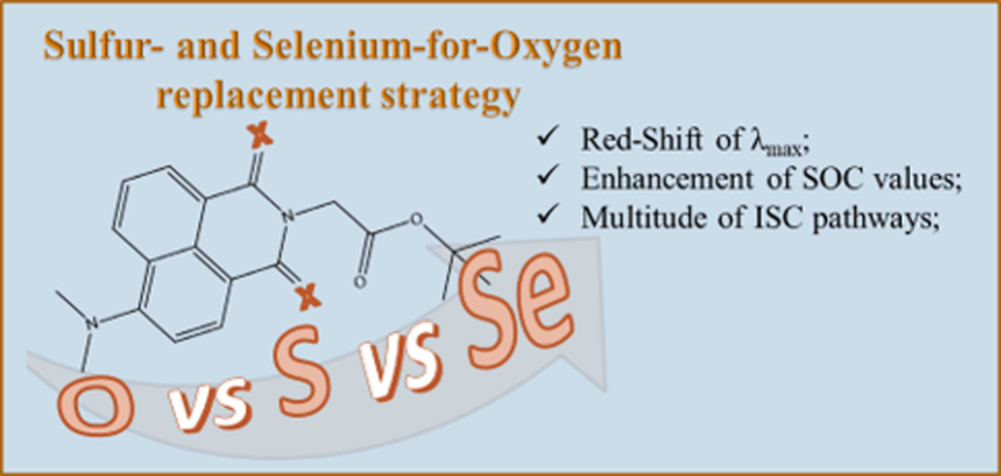

Thionation of carbonyl groups of known dyes is a rapidly emerging strategy to propose an advance toward heavy-atom-free photosensitizers to be used in photodynamic therapy (PDT). The sulfur-for-oxygen replacement has recently proved to enhance the singlet oxygen quantum yield of some existing fluorophores and to shift the absorption band at longer wavelengths. Drawing inspiration from this challenging evidence, the effect of both sulfur- and selenium-for-oxygen replacement in the skeleton of the oxo-4-dimethylamino-1,8-naphthalimide molecule (DMN) has been analyzed by means of a DFT study. The thio- and seleno-derivatives (SDMN and SeDMN, respectively) have been shown to offer the possibility to access a multitude of ISC (intersystem crossing) pathways involved in the triplet deactivation mechanisms with a consequent enhancement of the singlet oxygen production, also arising from the change of orbital type involved in the radiationless ^1^nπ* → ^3^ππ* transitions. Moreover, the change in nature from a ^1^ππ* to a ^1^nπ* observed in the SeDMN has been revealed to be crucial to reach more clinically useful regions of the spectrum suggesting that the selenium-for-oxygen replacement can be proposed as a strategy to achieve more suitable PDT agents while proposing an advance toward heavy-atom-free PSs.

## Introduction

Photodynamic therapy (PDT) is an approved noninvasive medical treatment used against a consistent number of diseases^1−4^ among which are different kinds of cancer,^5−7^ bacterial, viral and fungal infections,^8,9^ dental caries,^10^ rheumatoid arthritis,^11^ cardiovascular disorders,^12^ and cutaneous manifestations.^13^ Compared to chemotherapy, its use in anticancer therapy produces less side effects, and its high selectivity is enhanced by an invoked immune response which causes a mixture of apoptotic and necrotic cell death.^6,7^ For these reasons, basic and applied research aimed at proposing new drugs for PDT treatment has increased considerably over the last three decades,^14−24^ also with the aim of overcoming some of the most important limitations of current PDT, namely hypoxia and poor light penetration, focusing in particular on metallic dyes to abandon the use of traditional tetrapyrrolic structures.^16,24^

Light, oxygen, and a photosensitizer (PS) acting as a pro-drug are required for the clinical application of the therapy. The PS administration and localization in target tissue is followed by irradiation with a light source of proper wavelength to penetrate deeply in the tissue. The so-called therapeutic window that allows the treatment of deeper-seated tumors is limited at shorter wavelengths by the absorption properties of several skin chromophores and at longer wavelengths by water absorption, so that it is comprised between about 500 and 900 nm. Light irradiation triggers the photodynamic process, that is the photogeneration of singlet oxygen through an energy transfer process between the populated triplet state of the PS and the ground state oxygen present in the tissue (Type II photoreaction). On the other hand, excited triplet (T_1_) quenching mechanism can also include direct photoinduced electron transfer processes leading to other ROS (radical oxygen species) generation, among which the superoxide is one of the most important for its peculiar biochemical metabolism. Although their involvement in biomolecule degradation and tissue damage is well documented, Type II photoreactions are generally considered the predominant PDT mechanism due to the ability of singlet oxygen to target crucial unsaturated lipids, amino acid side chains and nucleic acids bases. Such a mechanism is likely to occur whether the PS has low fluorescence yield and if a significant intersystem spin crossing ensures population of a triplet excited state with energy higher than that required to promote the ^3^Σg → ^1^Δg oxygen transition (0.98 eV). Indeed, according to the Fermi golden rule the efficiency of the ISC (*k*_ISC_) mainly depends on the Δ*E*_S1-Tj_ energy gaps and on the spin–orbit coupling constant (SOCs) values.^25^

Accordingly, a good PDT photosensitizer must possess appropriate singlet–triplet energy separation and large singlet–triplet spin–orbit couplings to ensure an efficient ISC. It has been previously shown as SOC values computed for the approved drug Foscan (5,10,15,20-tetrakis(*m*-hydroxyphenyl)chlorin)^26^ are quite small (2.4 × 10^–1^ cm^–1^)^27^ but are still sufficient to trigger the energy transfer process. The insertion of a heavy atom in the molecular structure considerably increases SOCs and generally red shifts the absorption band.^26−31^ This positive effect is however countered by the occurrence of toxic effects generally due to their enhanced dark cytotoxicity. For this reason, sulfur or other atoms naturally present in tissues are being chosen as preferential heavier atoms to be inserted into potential drugs structures for PDT. Recent experimental works pointed out how the thionation of carbonyl groups of a series of known dyes increases the possibility to use them in PDT.^32−34^ Since then, the sulfur-for-oxygen replacement in existing fluorophores is rapidly emerging as strategy to enhance the singlet oxygen quantum yield and to shift the absorption band at longer wavelengths,^32^ while proposing an advance toward heavy-atom-free PSs.

**Figure 1:**
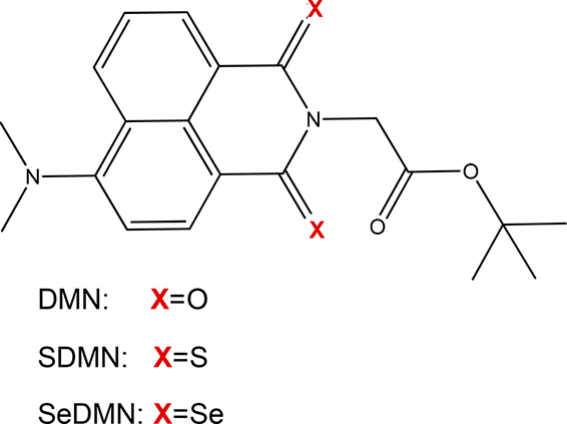
Molecular structures of DMN, SDMN, and SeDMN.

Drawn inspiration from that evidence, we decided to undertake a DFT study analyzing the effects of a single-atom replacement in the skeleton of the oxo-4-dimethylamino-1,8-naphthalimide molecule (DMN, Figure 1), in particular analyzing sulfur-for-oxygen (SDMN, Figure 1) and selenium-for-oxygen replacements (SeDMN Figure 1) with a resulting improvement of those photophysical properties crucial to propose these systems as drugs in PDT. Along with absorption properties and characterization, singlet–triplet energy gaps as well as SOCs values are herein provided. On the basis of these results, the possible deactivation paths are also predicted.

## Computational Details

Geometry optimizations and computations of excitation energies have been performed by using the hybrid Becke three-parameter exchange functional^35^ and the Lee–Yang–Parr correlation functional^36^ (B3LYP) coupled with the 6-31+G(d,p) basis set. Grimme dispersion corrections for nonbonding interactions have been included, applying an atom pairwise additive scheme (DFT-D3)^37^ method. In all computations, the solvent effects have been estimated using the IEFPCM continuum solvation model^38^ as implemented in Gaussian 16 code,^39^ considering implicit dimethyl sulfoxide (DMSO, ε = 46.826). Absorption spectra were obtained in DMSO as vertical electronic excitations on the ground-state structures at the TD-DFT/B3LYP/6-31+G(d,p) level of theory. The spin–orbit couplings (SOCs) were defined as
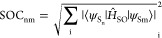
where *i* = *x*, *y*, *z*. *Ĥ*_*SO*_ is the spin–orbit Hamiltonian. They were obtained by using the atomic-mean field approximation^40^ as implemented in the DALTON code^41^ by using the B3LYP functional and the cc-pVDZ basis set for all the atoms.

This computational protocol has been previously used for the computations of a series of photophysical properties in a series of organic and inorganic systems.^19−24,42,43^

## Results and Discussion

**Figure 2:**
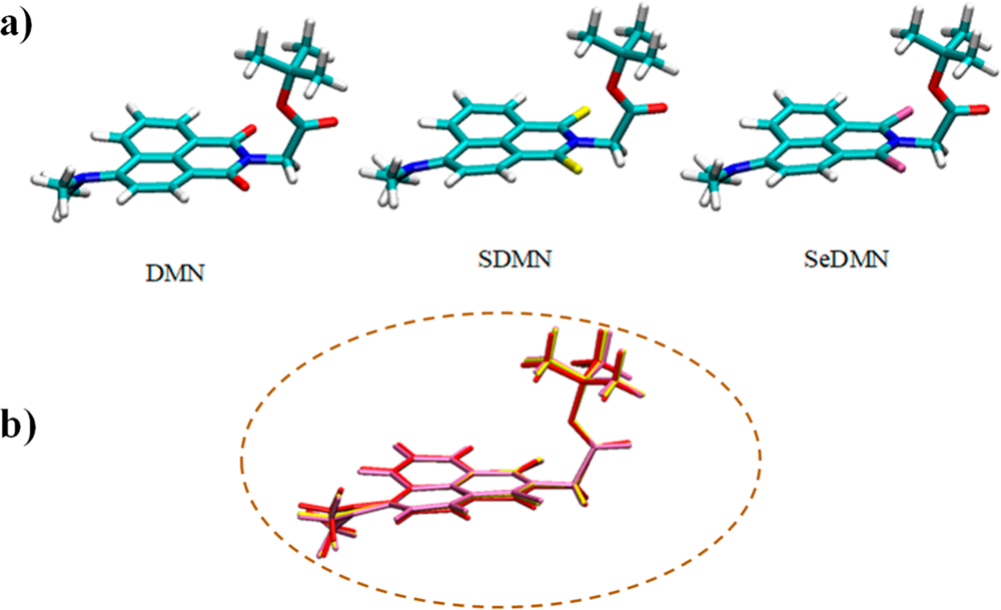
(a) Optimized geometries and (b) Superimposition of DMN, SDMN, and SeDMN in DMSO environment at the B3LYP/6-31+G(d,p) level of theory

Ground state optimized structures reveal that the sulfur- and selenium-for-oxygen substitution processes just slightly affects the main geometrical parameters of the dyes, as shown in Figure 2 and Table S1.

The peripheral ester groups lie perpendicularly to the naphthalimide moiety in all cases while the planarity of the rigid naphthalimide moiety decreases upon oxygen-replacement, being twisted by up to 4° with respect to the plane. The most significant difference between the investigated compounds concerns the C–X distances (Table S1) that, as expected, increase going down the chalcogen group with the longest value found for the SeDMN derivative. Main geometrical parameters computed for DMN, SDMN, and SeDMN are reported in the Supporting Information (Tables S1–S3).

**Figure 3:**
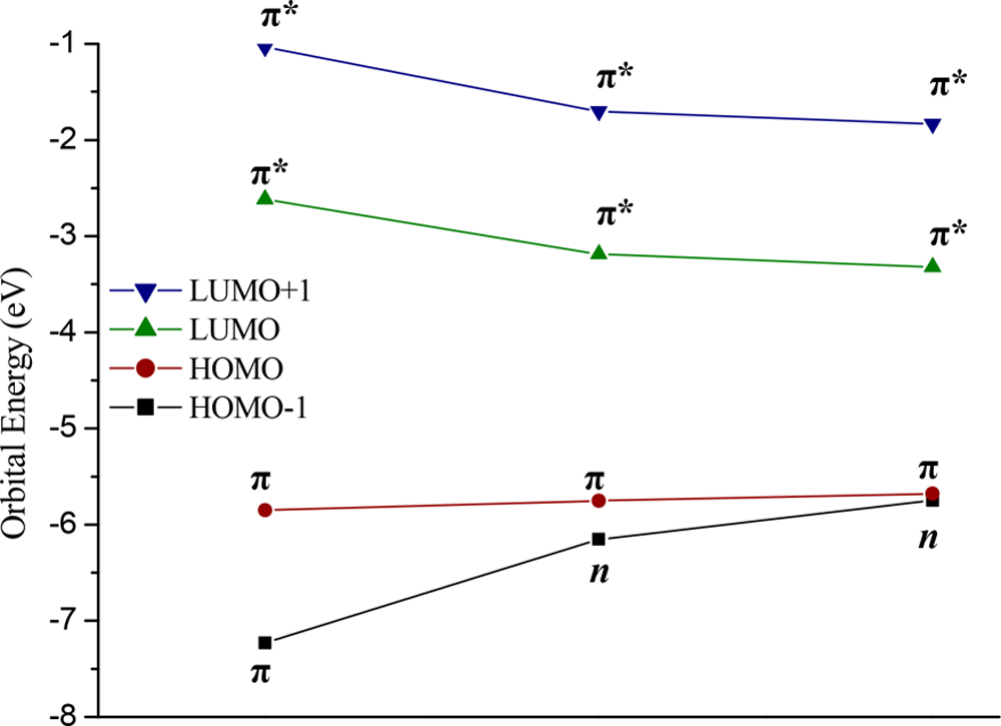
Calculated frontier orbital energies and H–L gaps (eV) for DMN, SDMN, and SeDMN compounds.

Inspection of the frontier orbitals allows one to detect an interesting drop in energy of the LUMO and LUMO+1 orbitals upon oxygen replacement with a consequent reduction of the HOMO–LUMO gaps in SDMN and SeDMN. By sharp contrast, it can be observed an increase of the HOMO–1 energy arising from a π (DMN) vs n nature change observed in the thio- and seleno- derivatives. The HOMO energy is much less affected by the single atom replacement since its π character is kept unchanged along the series. (See Figure 3 and Figure S1.)

**Figure 4:**
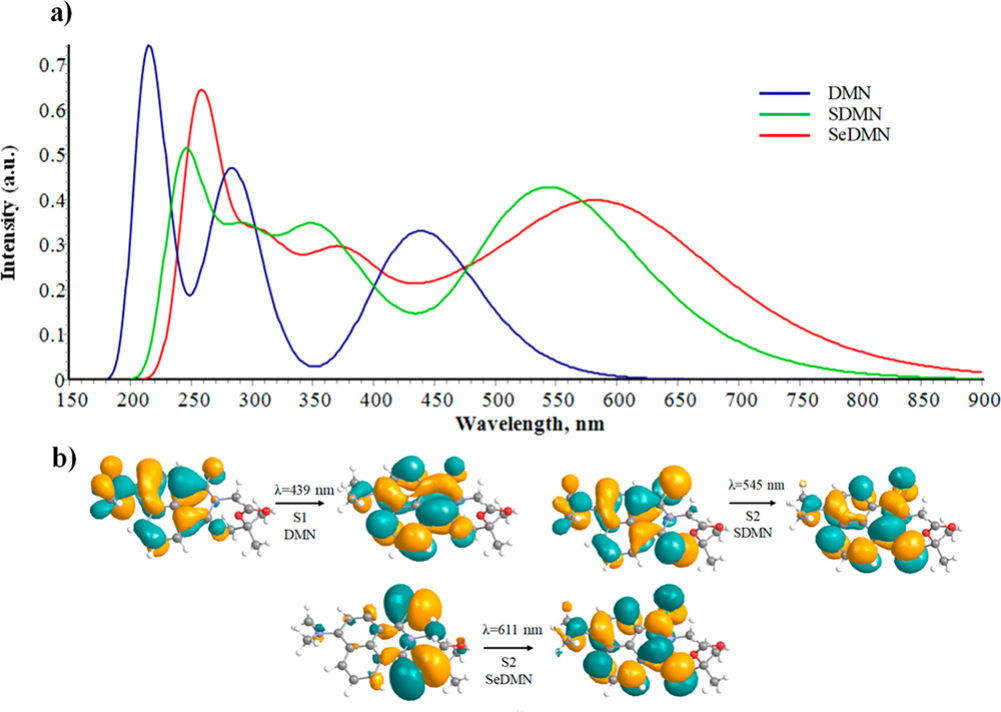
(a) Computed absorption spectra of DMN, SDMN and SeDMN in DMSO environment at the at the B3LYP/6-31+G(d,p) level of theory. (b) Natural transition orbitals (NTOs) characterizing the lowest energy transition for each compound. More details can be found in the Supporting Information.

A direct consequence of the reduction of HOMO–LUMO gap is the observed batochromic shift of the maxima absorption band. Indeed, a significant red shift characterizes the thio-based compound, and it is even more pronounced in the seleno-derivative. Indeed, in agreement with the experimental data (431 nm),^32^ the DMN compound shows an intense band centered at 439 nm (S_1_), clearly π → π* in nature as revealed by the natural transition orbitals involved (See Figure 4).

The sulfur–oxygen replacement produces a red shift of the lowest energy band, experimentally located at 604 nm. In this case, the S_1_ state computed at 593 nm has n → π* character and negligible oscillator strength, explaining the low fluorescence yields of the thio-derivative,^32^ while the π → π* S_2_ state can be directly populated and it is found at 545 nm (see Figure 4 and Figure S2).

The lowest energy band computed for SeDMN is further shifted at longer wavelengths. In this case, both S_1_ (741 nm) and S_2_ (611 nm) have n → π* character. Even in this case, the S_1_ state is not bright while S_2_ is the lowest energy populated state generating the intense band shown in Figure 4 (see also Figure S2).

It appears clear that the change in nature from π → π* to a n → π* is crucial to reach more clinically useful regions of the spectrum and the selenium-for-oxygen substitution can be proposed as a promising strategy. For SeDMN also, the S3 state can be populated under irradiation (588 nm) and the involved π → π* transition contributes to the broad band in the red region of the spectra. (Figure S2)

**Figure 5:**
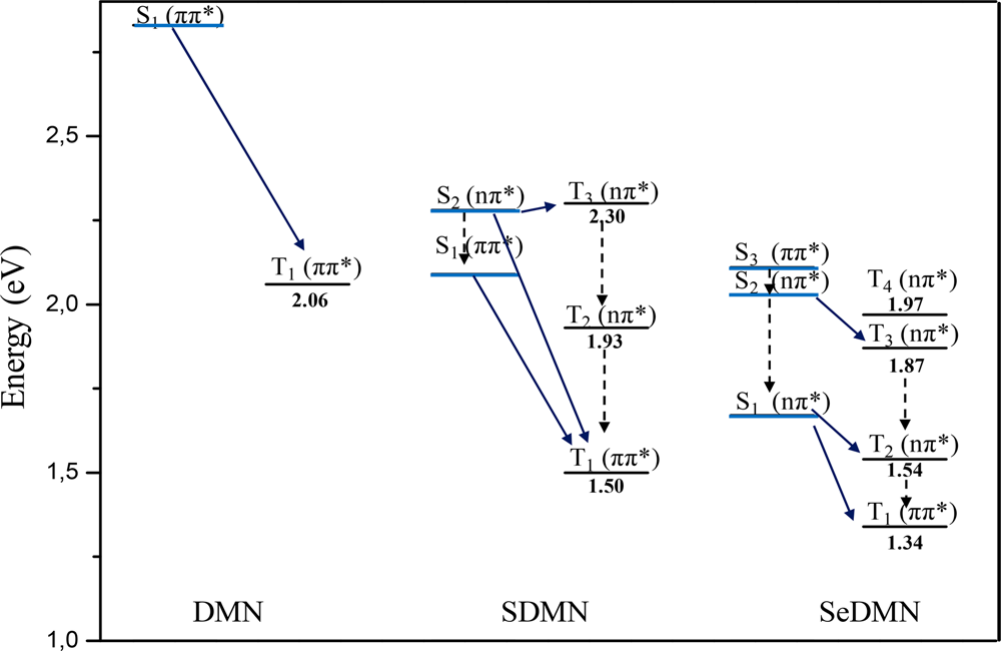
Singlet and triplet energies in the FC region and the proposed deactivation mechanism for DMN, SDMN, and SeDMN in DMSO.

Inspection of the triplet states in the FC region, allows to identify three and four triplet states lying below S_2_ in SDMN and S_3_ in SeDMN, respectively, so that the populated states could relax through at least four or six electronic states in the thio- and seleno-derivatives (Figure 5 and Table S2). Computed SOC values show strongly coupled states likely to give rise to efficient ISC to the triplet manifold, for SDMN and for SeDMN especially. Compared with DMN, for which a very low value has been obtained for the only accessible S_1_-T_1_ deactivation channel (0.5 cm^–1^), an enhancement of several orders of magnitude is obtained through a single atom replacement (127.6 and 833.3 cm^–1^, for SDMN and SeDMN respectively). The significant increase observed in the S_1_–T_1_ coupling for SeDMN is in agreement with the El Sayed rules^44^ since the radiationless transition involves a change of orbital type ^1^nπ* → ^3^ππ*.

For the thionated compound, even the S_2_–T_1_ coupling is characterized by high SOC values indicative of a possible role in the hole deactivation channel. Also in this case, the transition involves a change of orbital nature ^1^nπ* → ^3^ππ*. Nevertheless, the highest value is obtained between the almost isoenergetic S_2_ and T_3_, for which the large computed SOC reveal a strong coupling in the FC region and is indicative of a possible efficient ISC mechanism.

In the case of SeDMN, the highest SOC values (Table 1) are obtained for the S_1_–T_2_ channel, despite no observed change in the nature of the orbitals, and for the S_2_ (^1^nπ*)–T_3_(^3^ππ*) channel, also characterized by a small singlet–triplet splitting Δ*E*_S-T-_ The deactivation from S_3_ can also play a role, either through internal conversion toward S_2_ or through direct deactivation to the triplet manifold, although with a smaller probability.

**Table 1:** Computed SOC Value (cm^–1^) and Singlet–Triplet Energy Gaps Δ*E*_S-T_ (eV) between States Involved in Possible Deactivation Channels

	DMN		SDMN		SeDMN	
	SOC	Δ*E*_S-T_	SOC	Δ*E*_S-T_	SOC	Δ*E*_S-T_
**|⟨Ψ**_***S***_**1**__**|*****Ĥ***_***so***_**|Ψ**_***T***_**1**__**⟩|**	0.5	0.87	127.6	0.47	833.3	0.41
**|⟨Ψ**_***S***_**1**__**|*****Ĥ***_***so***_**|Ψ**_***T***_**2**__**⟩|**			4.3	0.18	2371.2	0.15
**|⟨Ψ**_***S***_**2**__**|*****Ĥ***_***so***_**|Ψ**_***T***_**1**__**⟩|**			72.7	0.86	572.7	0.78
**|⟨Ψ**_***S***_**2**__**|*****Ĥ***_***so***_**|Ψ**_***T***_**2**__**⟩|**			23.4	0.37	1359.3	0.52
**|⟨Ψ**_***S***_**2**__**|*****Ĥ***_***so***_**|Ψ**_***T***_**3**__**⟩|**			146.0	0.02	2552.1	0.21
**|⟨Ψ**_***S***_**3**__**|*****Ĥ***_***so***_**|Ψ**_***T***_**1**__**⟩|**					1476.5	0.84
**|⟨Ψ**_***S***_**3**__**|*****Ĥ***_***so***_**|Ψ**_***T***_**2**__**⟩|**					129.4	0.58
**|⟨Ψ**_***S***_**3**__**|*****Ĥ***_***so***_**|Ψ**_***T***_**3**__**⟩|**					334.1	0.27

On the basis of these results, it emerges that the insertion of selenium produces a multitude of ISC pathways involved in the triplet deactivation mechanisms, even more pronounced than upon thionation, due to the possibility to efficiently populate two singlet states with different nature and close in energy, contributing to enhance the singlet oxygen quantum yields.

Indeed, while for DMN just one S_1_–T_1_ channel can be proposed, for SDMN both S_1_ and S_2_ can play roles in the deactivation channels toward T_1_ and more than one triplet states can be involved in pathways from S_1_, S_2_, and even S_3_ for SeDMN.

Taking into account the Kasha rules^45^ and the possibility of faster interconversion (IC) processes compared with the intersystem crossings, the possible deactivation routes can be summarized as
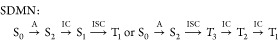

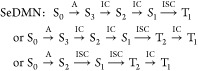


The available experimental O_2_ quantum yields negligible for DMN and almost equal to 1 for SMND^32^ agree well with our findings and lead us to conclude that results concerning the possible use of selenium are encouraging and deserve further exploration.

## Conclusions

The effect of both sulfur- and selenium-for-oxygen replacement in the skeleton of the oxo-4-dimethylamino-1,8-naphthalimide molecule has been analyzed by means of a DFT study. The thio- and seleno-derivatives allow one to reach longer absorption wavelengths and significantly enhance the possibility of ISC mechanisms toward the triplet manifold. Actually, the single-atom-substitution offers the possibility to access a multitude of ISC pathways involved in the triplet deactivation mechanisms characterized by significant large SOC values arising from the change of orbital type involved in the radiationless ^1^nπ* → ^3^ππ* transitions. Moreover, the change in nature from a ^1^ππ* to a ^1^nπ* observed in the SeDMN has proved to be an effective strategy to reach more clinically useful regions of the spectrum and to pave the way to achieve more suitable heavy-atom-free PDT agents.

## Supporting Information Available

Main geometrical parameters; computed main singlet absorption wavelengths; plots of HOMO, HOMO–1, HOMO–2, HOMO–3, LUMO, and LUMO+1 molecular orbitals; occupied (NTO o) and virtual (NTO (v) natural transition orbitals involved in the lowest singlet–singlet transitions and of the lowest lying triplet states; and *xyz* coordinates of the optimized structures, for the investigated compounds DMN, SDMN and SeDMN (PDF)
