# Supplementary material for: Chalcogen Effects in the Photophysical Properties of Dimethylamino-1,8-naphthalimide Dyes Revealed by DFT Investigation
Source: J Phys Chem A. 2022 Jul 27;126(31):5167–72. doi: 10.1021/acs.jpca.2c03950 (PMC9376948; doi:10.1021/acs.jpca.2c03950)
Supplement: Supplementary file 1 — jp2c03950_si_001.pdf [file jp2c03950_si_001.pdf]

Supporting Information

Chalcogen Effects in the Photophysical Properties of Dimethylamino-1,8-naphthalimide Dyes Revealed by DFT Investigation

*Marta Erminia Alberto, Bruna Clara De Simone, Tiziana Marino, Marirosa Toscano, Nino Russo**

Dipartimento di Chimica e Tecnologie Chimiche, Università della Calabria, Rende (CS), Italy.

Table S1. Main geometrical parameters computed for DMN, SDMN and SeDMN, according to the definition given in the picture

	DMN	SDMN	SeDMN
d	1.234	1.688	1.865
d_1	1.398	1.392	1.387
α	123.2	122.0	121.4
β	117.2	117.5	117.8
δ	177.7	175.5	173.8

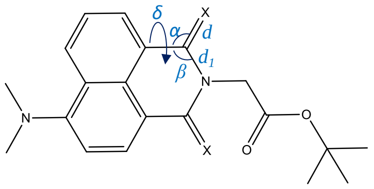

Table S2. Computed main singlet absorption wavelengths above 400 nm (λ)**,** oscillator strength (f) and main configuration, in DMSO environment at B3LYP/6-31+G(d,p) level of theory. Energy of triplet states lying below the bright singlet states.

	**DMN**				**SDMN**				**SeDMN**			
	eV	λ	%	f	eV	λ	%	f	eV	λ	%	f
S1	2.83	439	H→L 98%	0.331	2.09	593	H-1→L 97%	0.002	1.67	741	H-1→L 89%	0.001
S2					2.28	545	H→L 97%	0.420	2.03	611	H-2→L57%	0.121
S3									2.11	588	H→L 54%	0.252
T1	2.06	601	H→L 96%		1.50	826	H→L 98%		1.34	927	H→L 94%	
T2					1.93	642	H-1→L 95%		1.54	806	H-1→L 92%	
T3					2.30	539	H-2→L 74%		1.87	662	H-2→L 83%	
T4									1.97	628	H-2→L 80%	

Figure S1. Plots of HOMO, HOMO-1, HOMO-2, HOMO-3, LUMO and LUMO+1 Molecular Orbitals for DMN, SDMN and SeDMN, computed in DMSO, at B3LYP/6-31+G(d,p) level of theory

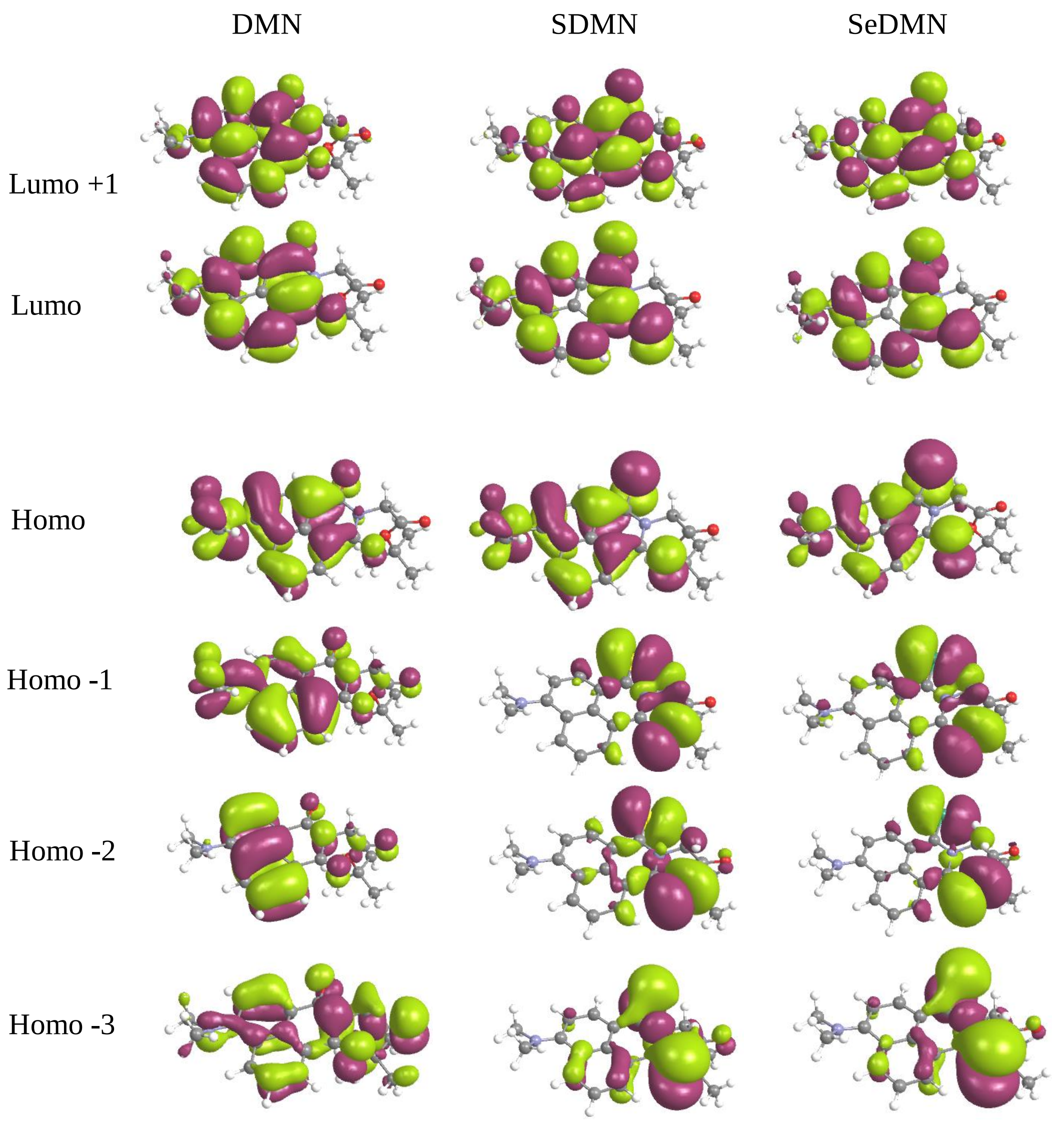

Figure S2. Occupied (NTO o) and virtual (NTO v) Natural Transition Orbitals involved in the lowest singlet-sinlget transitions DMN, SDMN and SeDMN, computed in DMSO, at B3LYP/6-31+G(d,p) level of theory

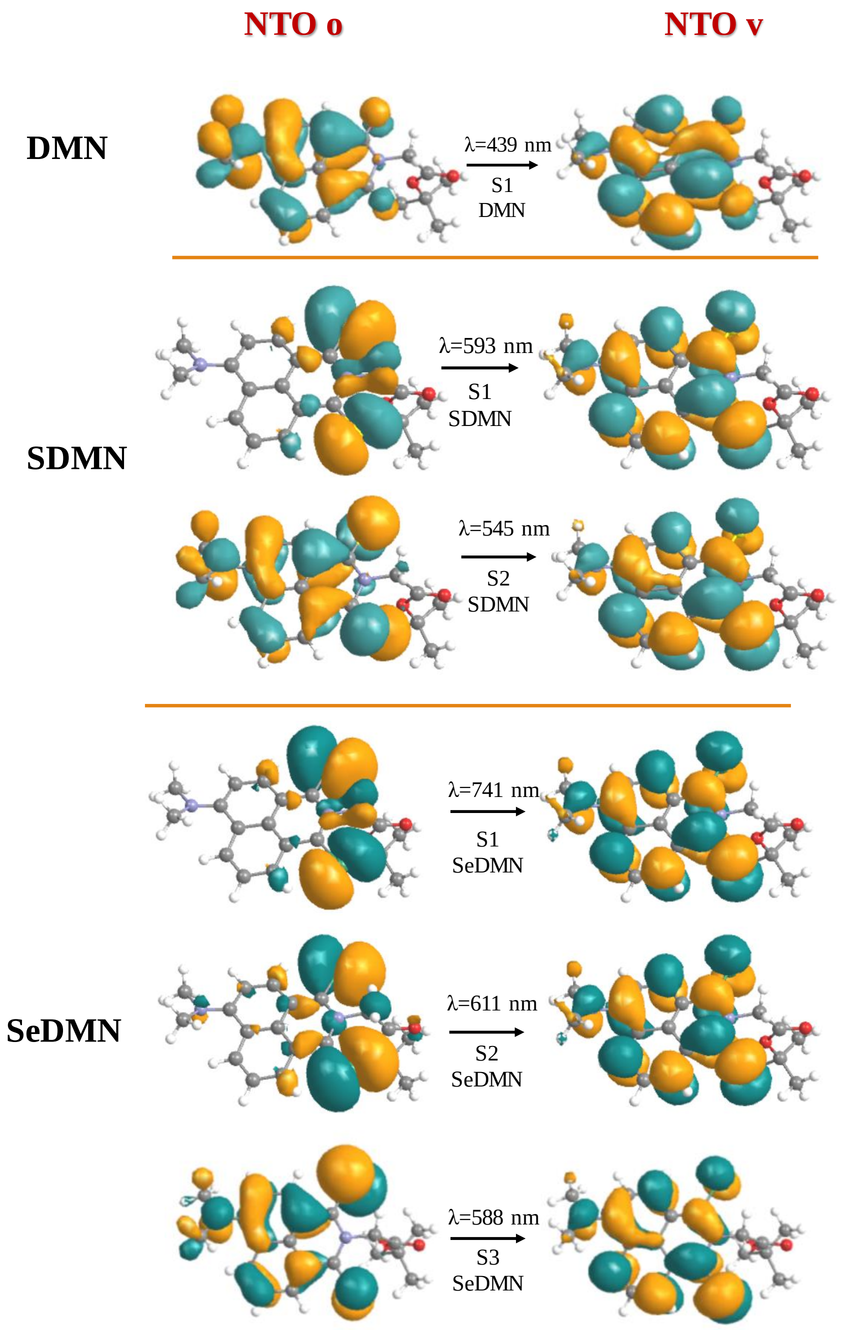

Figure S3. Occupied (NTOo) and virtual (NTOv) Natural Transition Orbitals of the triplet states lying below S1 for a) DMN and below S2 state for b) SDMN and c) SeDMN compounds.

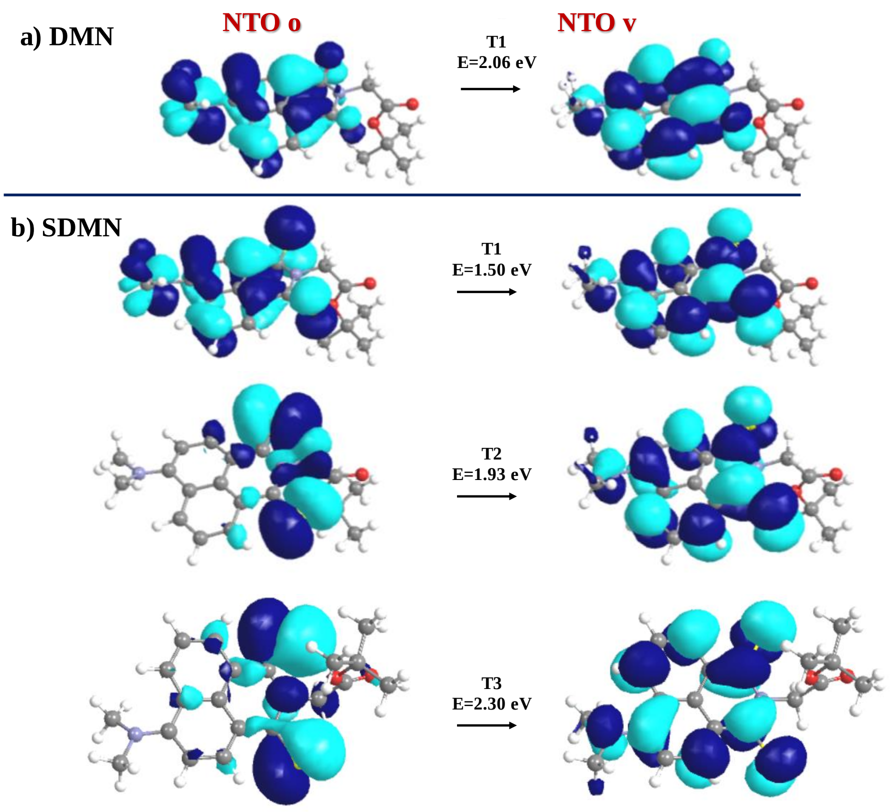

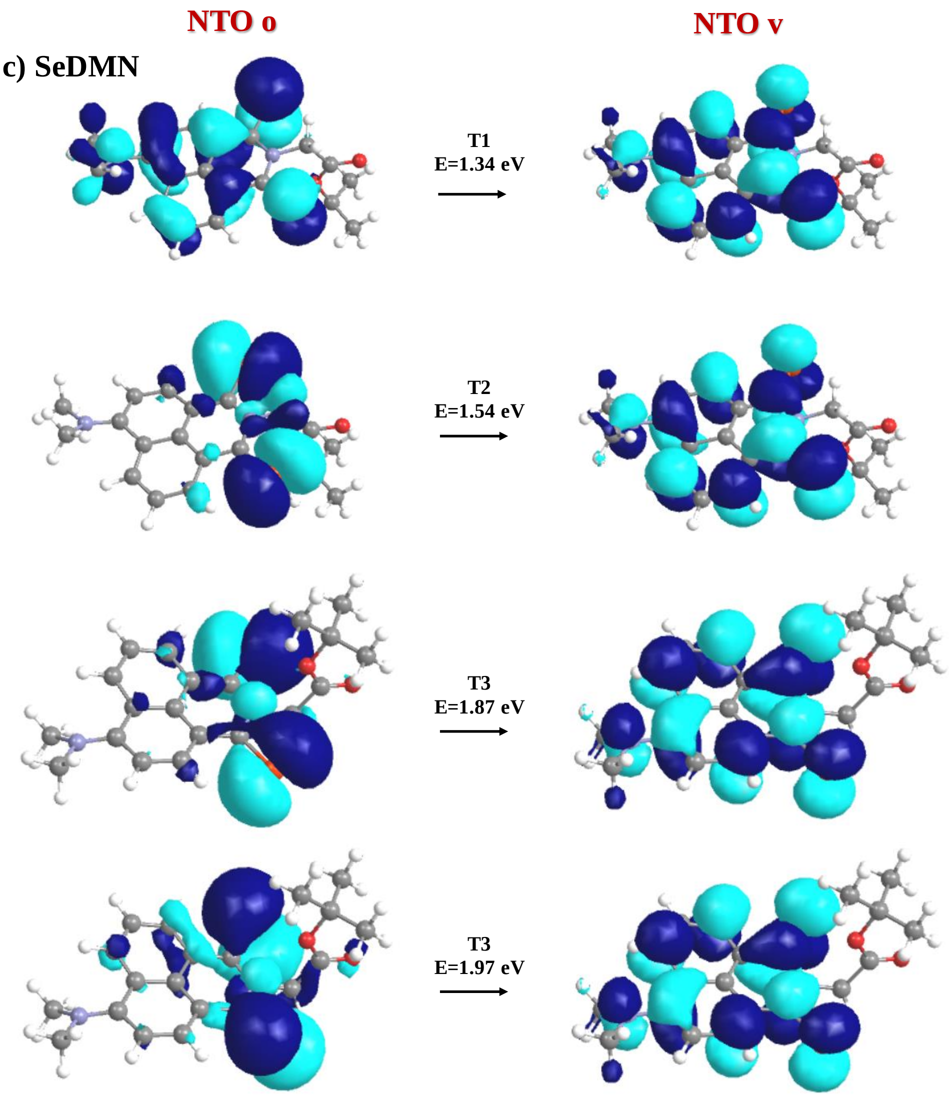
NTO o
NTO v
c) SeDMN
T1
E=1.34 eV
T2
E=1.54 eV
T3
E=1.87 eV
T3
E=1.97 eV

Table S3: xyz Coordinates of the optimized geometries of a) DMN, b) SDMN, c) SeDMN

a) DMN

48

C	-5.951650	-4.483380	-0.861568
C	-4.626862	-4.250316	-1.234584
C	-3.989779	-3.045380	-0.950429
C	-4.686687	-2.059002	-0.202743
C	-6.034433	-2.288009	0.222324
C	-6.708361	-3.504479	-0.195912
C	-2.617387	-2.818136	-1.400182
C	-4.031153	-0.854585	0.171884
C	-2.653222	-0.591324	-0.286200
C	-4.663982	0.070611	0.991746
C	-5.953140	-0.191821	1.481632
C	-6.619923	-1.347454	1.106374
H	-7.598043	-1.546193	1.526430
H	-6.424198	0.505808	2.166062
H	-6.413969	-5.416796	-1.155614
H	-4.087143	-5.008984	-1.791571
H	-4.138060	0.977607	1.268858
N	-2.040240	-1.574253	-1.068792
C	-0.682743	-1.297825	-1.526011
H	-0.461774	-1.989583	-2.340469
H	-0.625352	-0.275828	-1.899931
C	0.415582	-1.462862	-0.477262
O	0.024252	-2.187441	0.567788
O	1.526188	-0.990303	-0.657547
N	-8.042618	-3.710605	0.101683
C	-8.582371	-5.066978	0.048276
H	-7.904036	-5.767023	0.539808
H	-9.533744	-5.076638	0.586010
H	-8.769101	-5.407290	-0.981639
C	-9.054941	-2.691850	-0.218079
H	-9.734836	-2.538986	0.626430
H	-8.589657	-1.743509	-0.478450
H	-9.645658	-3.025340	-1.082038
C	0.928833	-2.529037	1.706867
C	0.012517	-3.345144	2.619447
H	0.566936	-3.662794	3.507321
H	-0.845325	-2.747992	2.942653
H	-0.355172	-4.237062	2.103471
C	1.390116	-1.244760	2.399463
H	2.047697	-0.655674	1.758704
H	0.529343	-0.633159	2.687073
H	1.937597	-1.509643	3.309605
C	2.093664	-3.379700	1.195488
H	1.720918	-4.252103	0.649662
H	2.754414	-2.806567	0.543428
H	2.674839	-3.737621	2.051258
O	-2.034675	0.440280	-0.010027
O	-1.962657	-3.641561	-2.047658

b) SDMN

48

C	-5.952899	-4.594268	-0.795095
C	-4.624478	-4.377564	-1.105841
C	-3.975611	-3.150163	-0.864703
C	-4.709842	-2.137797	-0.186783
C	-6.078994	-2.352508	0.189956
C	-6.753439	-3.570246	-0.236039
C	-2.607962	-2.935205	-1.263056
C	-4.063702	-0.922828	0.176558
C	-2.698276	-0.660538	-0.278258
C	-4.738544	0.002731	0.981399
C	-6.028410	-0.257786	1.442601
C	-6.688669	-1.414714	1.050303
H	-7.675582	-1.608175	1.448767
H	-6.515132	0.441685	2.113684
H	-6.397689	-5.540660	-1.071981
H	-4.065180	-5.166301	-1.594325
H	-4.230312	0.915708	1.265619
N	-2.055396	-1.666183	-0.994453
S	-1.914438	0.800724	0.039818
S	-1.683371	-4.134902	-2.027558
C	-0.705278	-1.373778	-1.491549
H	-0.481231	-2.097643	-2.275388
H	-0.690134	-0.369135	-1.909187
C	0.420789	-1.467355	-0.467019
O	0.074280	-2.139218	0.627002
O	1.520779	-1.000930	-0.715768
N	-8.097107	-3.748669	-0.088634
C	-8.678041	-5.089674	-0.181798
H	-8.055164	-5.815781	0.342232
H	-9.657025	-5.069169	0.300855
H	-8.811863	-5.407773	-1.224524
C	-9.084578	-2.675115	-0.268048
H	-9.642576	-2.481621	0.654143
H	-8.606192	-1.757244	-0.604151
H	-9.795726	-2.989782	-1.039307
C	1.019308	-2.405199	1.752921
C	0.142830	-3.177234	2.740097
H	0.729969	-3.439490	3.625056
H	-0.710400	-2.570846	3.058225
H	-0.231751	-4.099963	2.287203
C	1.487029	-1.078980	2.356363
H	2.116992	-0.520591	1.662485
H	0.628526	-0.460614	2.635682
H	2.066118	-1.286342	3.261861
C	2.177632	-3.271913	1.253831
H	1.798620	-4.178514	0.771972
H	2.809753	-2.730867	0.548238
H	2.790393	-3.572870	2.109558

c)SeDMN

48

C	-5.946777	-4.627962	-0.786566
C	-4.618920	-4.409221	-1.080504
C	-3.971153	-3.174212	-0.840582
C	-4.719398	-2.160892	-0.176384
C	-6.090539	-2.380697	0.190597
C	-6.760474	-3.598802	-0.246406
C	-2.608159	-2.952118	-1.215254
C	-4.079563	-0.940818	0.186287
C	-2.728959	-0.669964	-0.283227
C	-4.764361	-0.024902	1.001033
C	-6.050319	-0.295954	1.459640
C	-6.706706	-1.453026	1.054638
H	-7.693747	-1.652444	1.449689
H	-6.540987	0.395078	2.136679
H	-6.385061	-5.576769	-1.065382
H	-4.051609	-5.199610	-1.556912
H	-4.263426	0.889879	1.292100
N	-2.070795	-1.676437	-0.973716
C	-0.725081	-1.378921	-1.485930
H	-0.512657	-2.079980	-2.292808
H	-0.716750	-0.363881	-1.878292
C	0.419449	-1.497145	-0.483483
O	0.066540	-2.091206	0.651130
O	1.534952	-1.102201	-0.783174
N	-8.103406	-3.774924	-0.140009
C	-8.688963	-5.113861	-0.257301
H	-8.079800	-5.848398	0.270767
H	-9.676524	-5.092950	0.207052
H	-8.803610	-5.417455	-1.306071
C	-9.089324	-2.694226	-0.281422
H	-9.632388	-2.517525	0.653000
H	-8.612519	-1.772050	-0.608289
H	-9.812546	-2.994389	-1.046482
C	1.025047	-2.352300	1.766482
C	0.134606	-3.025752	2.811948
H	0.729947	-3.276437	3.694931
H	-0.676196	-2.358367	3.119133
H	-0.300837	-3.947595	2.414870
C	1.578677	-1.025123	2.290562
H	2.219003	-0.536854	1.554386
H	0.762173	-0.347503	2.558471
H	2.170019	-1.219025	3.191293
C	2.122701	-3.304355	1.285233
H	1.684017	-4.211314	0.857617
H	2.765389	-2.833541	0.539741
H	2.739620	-3.596095	2.141274
Se	-1.906838	0.977645	0.016015
Se	-1.558021	-4.300054	-1.993402
